# Prognosis and Failure Patterns of 11q13 Amplified Local Advanced Squamous Cell Carcinoma of the Head and Neck

**DOI:** 10.1002/cam4.71235

**Published:** 2025-09-20

**Authors:** Chang Jiang, Shengjin Dou, Yu Wang, Wen Jiang, Lulu Ye, Rongrong Li, Xiaolong Fu, Lin Zhang, Guopei Zhu

**Affiliations:** ^1^ Department of Radiation Oncology Shanghai Chest Hospital, Shanghai Jiao Tong University Shanghai China; ^2^ Department of Oral and Maxillofacial‐Head Neck Oncology Shanghai Ninth People's Hospital, Shanghai Jiao Tong University, School of Medicine Shanghai China; ^3^ Department of Oral Pathology Shanghai Ninth People's Hospital, Shanghai Jiao Tong University, School of Medicine Shanghai China

## Abstract

**Background:**

Amplification of 11q13 (FGF3/4/19, CCND1) is frequently observed in head and neck squamous cell carcinoma (HNSCC). However, there is a lack of research investigating 11q13 amplification as a prognostic marker for patients with locally advanced (LA) HNSCC who undergo postoperative radiotherapy (PORT).

**Materials and Methods:**

This retrospective study included consecutive patients of LA‐HNSCC who underwent radical surgical resection and PORT. The 11q13 amplification was tested by next‐generation Sequencing (NGS) or fluorescent in situ hybridization (FISH). Propensity score matching (PSM) was used to match the amplification and wild‐type groups. Univariate and multivariate analyses were conducted using Kaplan–Meier and Cox regression. Recurrence patterns and phenotypes in the amplification group were also assessed. Statistical analyses were performed using R software, with a *p*‐value of < 0.05 considered statistically significant.

**Results:**

A total of 70 patients were included (35 in the 11q13 amplification group and 35 in the wild‐type group). Patients with 11q13 amplification exhibited significantly worse disease‐free survival (DFS) (3‐year DFS: 21.0% vs. 52.6%; *p* < 0.0019) and overall survival (OS) (3‐year OS: 46.4% vs. 66.7%; *p* = 0.032) compared to wild‐type patients. The recurrence pattern in the amplification group showed an approximately equal proportion of local‐regional recurrence (LRR) and distant metastases (DM). The LRR predominantly occurred within the 60 Gy radiation field. Multivariate analyses revealed that 11q13 amplification significantly associated with worse DFS (*p* < 0.001) and OS (*p* = 0.007).

**Conclusion:**

LA‐HNSCC patients with 11q13 amplification exhibited significantly worse DFS and OS compared to wild‐type patients. The recurrence pattern in the 11q13 amplification group was primarily characterized by in‐field recurrences within the 60 Gy dose.

## 1 Introduction

Head and neck squamous cell carcinoma (HNSCC) is the sixth most common cancer worldwide, accounting for approximately 450,000 deaths annually [1]. At the time of diagnosis, 70%–80% of HNSCC cases are locally advanced, and the standard treatment typically involves surgery followed by postoperative radiotherapy and chemotherapy [2]. However, approximately 50% of patients experience recurrence or metastasis later [3]. Despite the improvements in survival observed with neoadjuvant therapies such as immune checkpoint inhibitors or targeted therapies, clinical trials indicate that about half of patients may not benefit from combination therapies [4]. Therefore, it is crucial to identify patients with poorer prognosis prior to treatment, enabling tailored approaches such as radiation dose escalation in recurrence‐prone areas or early incorporation of targeted therapies [5].

Historically, clinical and histopathological features such as multiple lymph node metastases, metastasis to zones IV/V, HPV‐positive, and extracapsular node extension (ENE) in lymph nodes have been recognized as poor prognostic indicators [6]. In the era of precision medicine, there is a growing need to develop genetic prognostic markers to better optimize molecular subtyping [6]. Fibroblast growth factors (FGF) mutations, including FGF amplification and fibroblast growth factors receptor (FGFR) mutations, are among the most frequent genetic alterations in HNSCC [7, 8]. FGF2 has previously been identified as a prognostic marker for HNSCC [9]. Amplification in FGF3, FGF4, and FGF19 has a higher incidence in HNSCC, with frequencies of 22.9%, 21.2%, and 22.6%, respectively [2].

The human FGF gene family consists of 22 members (FGF1–FGF23, excluding FGF15) [10]. FGF3 and FGF4 bind to FGFR1–3, initiating downstream signaling that promotes cell migration, proliferation, and survival [11, 12]. Additionally, FGFR4, a key gene in liver cancer, interacts with FGF19 in a heparin‐dependent and highly affinity‐specific manner [13]. Cell Cyclin D1 (CCND1) forms a complex with CDK4/6 and promotes cell transition from G1 to S phase [14]. CCND1 also promotes cell proliferation by regulating the expression of other genes, which is positively correlated with the immune escape ability of tumors [15]. Amplification of FGF3/FGF4/FGF19/CCND1 resides on the same amplicon on the long arm of the chromosome and they often exhibit synergistic amplification [8, 16, 17]. They can activate FGFR kinase, triggering downstream signaling pathways such as PI3K/Akt, STATs, or RAS/RAF/ERK, which play a crucial role in various biological processes, including organ development, tumor angiogenesis, tissue regeneration after injury, and embryogenesis [10, 18]. In April 2019, the Food and Drug Administration (FDA) approved Erdafitinib, an FGFR inhibitor, as the first treatment for urethral carcinoma [19]. However, there remains a significant gap in clinical data regarding the prognostic role of FGF3/4/19 in HNSCC patients.

Our previous study indicated that 11q13 amplification negatively correlated with programmed death‐1 (PD‐1) inhibitor therapy benefit in a retrospective clinical cohort [20]. The current study aims to investigate the predictive impact of 11q13 amplification in patients receiving radiation therapy and surgery for locally advanced (LA) HNSCC. Additionally, we explored recurrence patterns after postoperative radiotherapy (PORT). This study further investigates the relationship between disease‐free survival (DFS) and overall survival (OS) in relation to other clinical and histopathological features.

## 2 Materials and Methods

### 2.1 Enrollment and Follow‐Up

The patients enrolled in this study were treated between April 1, 2018, and October 31, 2023, at Shanghai Ninth People's Hospital. Eligible participants were aged 18–75 years, had undergone complete resection (either radical or extended), and were pathologically diagnosed with T1‐2N1‐N3M0 or T3‐4N0‐3M0 HNSCC according to the American Joint Committee on Cancer (AJCC) 8th staging criteria. All patients were required to have an Eastern Cooperative Oncology Group (ECOG) performance status of 0 or 1. Exclusion criteria included patients who received neoadjuvant therapy, had concurrent or sequential second primary cancers, or had uncontrolled active infections.

Clinical and pathological data were collected from patients during their treatment and follow‐up visits. After PORT, patients were generally followed up every 3 months during the first 2 years and every 6–12 months thereafter. Follow‐up visits were conducted through outpatient appointments and telephone follow‐ups. These visits included a review of the clinical history, physical examinations, head and neck magnetic resonance imaging (MRI) scans, computed tomography (CT) scans of the chest, and ultrasound of the abdomen.

### 2.2 Assessment and Definition

OS was defined as the time elapsed between the date of the surgery and the last follow‐up or the patient's death, with any death considered an event. DFS was defined as the time interval between the date of the surgery and the date of the first recurrence or the last follow‐up, with recurrence being handled as an event. The study also examined the loco‐regional recurrence (LRR) location and the pattern of treatment failure at the first presentation following PORT. Recurrence at the head and neck, as well as the head and neck lymph node drainage sites, was categorized as LRR. Failure at distant organs and other sites was classified as distant metastases (DM). The recurrence phenotype of LRR was further classified as either in‐field or out‐of‐field failure, depending on the correlation between the LRR site and the targeted volume of PORT.

### 2.3 Postoperative Chemoradiotherapy/Radiotherapy

All patients were immobilized using thermoplastic head and shoulder masks during treatment‐planning CT imaging. Clinical information, physical examination, and contrast‐enhanced CT and/or MRI were utilized to estimate the target volumes and radiation doses. Over a period of 6–7 weeks, postoperative intensity‐modulated radiation therapy (IMRT) was delivered 5 days a week, with a total dose of 60–66 Gy (200 cGy per fraction per day). The clinical target volumes (CTV) were categorized as follows: high‐risk target volumes were covered by CTV60 (60 Gy dose), low‐risk regions by CTV54 (54 Gy dose), and, at the discretion of the radiation oncologist, a CTV6 boost (6 Gy dose) was added for positive margins and/or ENE. To account for potential variations in treatment setup and internal organ movements, the planning target volume (PTV) was defined as the CTV plus a 5‐mm margin. Standardized departmental procedures were adhered to during the planning and administration of radiotherapy. For patients aged ≤ 65 years with positive margins, ENE, or multi‐level lymph node metastasis, concurrent cisplatin at a dose of 80 mg/m^2^ every 3 weeks was administered.

### 2.4 11q13 Amplification Test

In this study, 11q13 amplification was tested using either next‐generation sequencing (NGS) or CCND1 fluorescent in situ hybridization (FISH), as these two methods were found to be significantly correlated. Sequencing was conducted to assess FGF3/4/19 gene amplification in medical samples using the QIAgen DNA FFPE tissue kit (Germantown, MD, USA). Tumor genomic deoxyribonucleic acid (DNA) was extracted from formalin‐fixed, paraffin‐embedded (FFPE) tissue sections. This DNA was then used for targeted sequencing with a cancer‐related gene panel from Genecast Biotech (Wuxi, China). Genomic DNA was also isolated from the peripheral blood of participants using the QIAgen DNA Blood Micro Kit, with a matched control sample obtained for comparison. For each participant, 300 ng of genomic DNA was used to construct the sequencing library. Target regions were enriched using IDT library preparation kits (Integrated DNA Technologies, Coralville, IA, USA), and fragment libraries were created from samples that had been sheared by sonication. The paired‐end library was sequenced using the Illumina NovaSeq 6000 platform (San Diego, CA, USA) after amplification of the collected DNA. Bioinformatics analysis was performed using an in‐house program developed by Genecast Biotech.

CCND1 amplification was tested by FISH staining. This was done using complete slides of FFPE tumor specimens in accordance with the manufacturer's CCND1‐specific probe labeling instructions. Acquire images using the ZEISS Axioscan 7 Microscope Slide Scanner. CCND1 gene amplification typically appears as additional fluorescent signal spots in the nuclei. A certified pathologist interprets the results based on the distribution and number of fluorescent signals.

### 2.5 Statistical Considerations

The specific process for propensity score matching (PSM) between the amplification group and the wild‐type group is detailed in the supplement. The effectiveness of the intervention was assessed using chi‐square analysis, while logistic regression was employed to calculate the hazard ratio (HR) and 95% confidence interval (CI). DFS and OS were estimated using the Kaplan–Meier (KM) method. Differences in KM curves were assessed using the log‐rank test, with results considered statistically significant if *p* < 0.05. In multivariate analysis, the Cox proportional hazards model was used to evaluate predictors that were statistically significant (*p* < 0.05) or showed significance in univariate analysis. The relationship between mutation frequency, amplification factors, and survival was examined using Pearson correlation analysis, with *p* < 0.05 considered statistically significant. To investigate differences in baseline characteristics, *t*‐tests were employed. For all statistical analyses and the generation of heat maps for clinical features, statistical product and service solutions (SPSS) and the R programming language were used.

## 3 Results

### 3.1 Patient Characteristics

Between April 1, 2018, and October 31, 2023, a total of 35 patients were enrolled in each group after PSM: one group with 11q13 amplification and the other with 11q13 wild‐type. The clinical characteristics were well balanced between the two groups. The average age was 59 years (range: 29–76 years) in the 11q13 amplification group and 62 years (range: 44–76 years) in the wild‐type group. The gender distribution was 4 women to 31 men in both groups. Each group included 15 patients who received surgery and radiotherapy and 20 patients who underwent surgery with concurrent chemoradiotherapy (CCRT). Approximately half of the patients in each group had ENE and multi‐region lymph node metastasis. A detailed summary of the baseline characteristics of the study participants is presented in Table 1.

**TABLE 1:** Patients characteristic.

Characteristic	Mutation patients (%)	Wild type patients (%)	*p*
11q13 gene status			
11q13 amplification	35 (100)		
11q13 wild‐type	—	35 (100)	
Age			
< 60 years	23 (66)	22 (65)	0.80
≥ 60 years	12 (34)	13 (35)	
Gender			
Female	4 (12)	4 (12)	1.0
Male	31 (88)	31 (88)	
HPV status			
Positive	4 (11)	5 (14)	
Negative	31 (89)	30 (86)	
Tumor site			
Oropharynx	4 (12)	3 (8)	0.57
Carcinoma of the maxillary sinus	3 (8)	2 (6)	
Oral cavity	28 (80)	30 (86)	
Treatment			
S + RT	15 (43)	15 (43)	1.0
S + CCRT	20 (57)	20 (57)	
TNM			
III	8 (23)	4 (12)	0.21
IV	27 (77)	31 (88)	
Histological grade			
G1‐2	19 (54)	22 (63)	0.47
G3	16 (46)	13 (37)	
T‐category			
1–2	15 (43)	17 (49)	0.63
3–4	20 (57)	18 (51)	
N‐category			
0–2	15 (43)	12 (34)	0.46
3	20 (57)	23 (66)	
ENE			
Positive	20 (57)	20 (57)	1.0
Negative	15 (43)	15 (15)	
Multi‐regions ENE			
Positive	7 (20)	9 (26)	0.57
Negative	28 (80)	26 (74)	
IV/V			
Positive	7 (20)	6 (19)	0.76
Negative	28 (80)	29 (81)	
Multi‐regions lymph node metastasis			
Positive	20 (57)	19 (56)	0.81
Negative	15 (43)	16 (44)	

### 3.2 Survival Analysis

**FIGURE 1:**
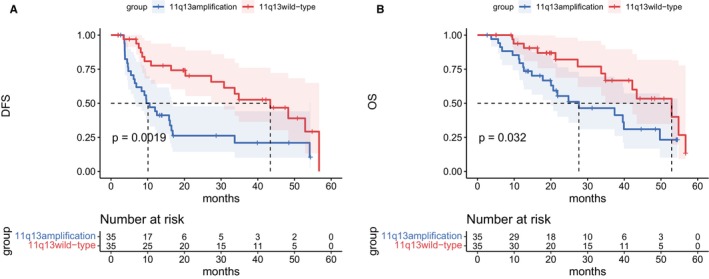
Clinical survival curves.

With a median follow‐up of 21 months, a total of 20 and 13 deaths were reported in the 11q13 amplification group and the 11q13 wild‐type group, respectively. The median OS was 27.6 months (range: 1.9–56.7 months) in the 11q13 amplification group and 52.9 months (range: 2.6–56.7 months) in the 11q13 wild‐type group. The 3‐year OS rates were 46.4% and 66.7%, respectively, with a significant difference between the two groups (HR, 2.06; 95% CI, 1.037–4.119; two‐sided log‐rank *p* = 0.032; Figure 1B). The median DFS was 10.1 months (range: 1.3–100.2 months) in the 11q13 amplification group and 43.4 months (range: 1–137.1 months) in the 11q13 wild‐type group. The 3‐year DFS rates were 21.0% and 52.6%, respectively, with a significant difference between the two groups (HR, 2.445; 95% CI, 1.327–4.507; two‐sided log‐rank *p* = 0.0019; Figure 1).

### 3.3 Recurrence Pattern and Phenotype of 11q13 Amplification Cohort

**FIGURE 2:**
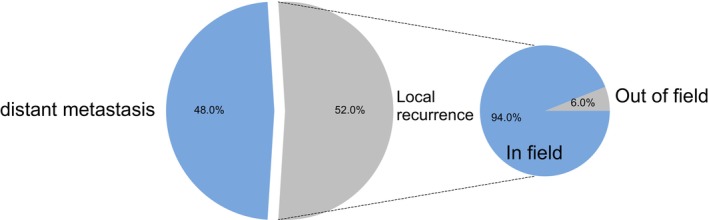
Recurrence pattern of 11q13 amplified group and wild‐type group. A. Recurrence model of 11q13 amplified group; B. Recurrence model of 11q13 wild‐type group.

A total of 31 patients ultimately developed recurrence in the 11q13 amplification group. A total of 16 (52%) patients developed LRR: 13 were isolated, and 3 were synchronous with DM. A total of 18 patients experienced DM: 15 (48%) were isolated, and 3 were synchronous. The recurrence loci were categorized based on their central placement as in‐field and out‐of‐field. Among the 16 LRR occurrences, 15 (94%) in the 11q13 amplified group were dominated by an in‐field recurrence within the 60Gy radiation volume (Table 2). Fourteen patients experienced a total of 20 recurrence sites in the lymph node area. Level III (20%, 4/20) was the most frequently affected site, followed by level Ib (15%, 3/20), level VII (15%, 3/20), and level IX (15%, 3/20) (Figure 2).

**TABLE 2:** Relationship between LRR patterns and radiotherapy exposure fields.

No	Primary	Failure site	Recurrence phenotype
1	Oral cavity	#I b,III	In‐field (60Gy)
2	Oral cavity	#III	In‐field (60Gy)
3	Oral cavity	#VIII,IX	In‐field (60Gy)
4	Oropharynx	VII a	Out‐of‐field (60Gy)
5	Oral cavity	Tumor bed	In‐field (60Gy)
6	Oral cavity	VI a	In‐field (56Gy)
7	Oral cavity	IX	In‐field (60Gy)
8	Oral cavity	III	Margin (60Gy)
9	Oral cavity	IX	In‐field (60Gy)
10	Oral cavity	VII a	In‐field (60Gy)
11	Oropharynx	II,VII a	In‐field (60Gy)
12	Oral cavity	I b	In‐field (60Gy)
13	Oral cavity	I b	In‐field (60Gy)
14	Oropharynx	VIII	In‐field (60Gy)
15	Oral cavity	II,VII a	In‐field (60Gy)
16	Oral cavity	I b	In‐field (60Gy)

### 3.4 Univariate Analysis and Multivariate Analysis of Survival

In a subgroup analysis of DFS, both univariate and multivariate analyses showed significance differences in the treatment (uni‐*p* = 0.004, multi‐*p* < 0.001), histological grade (uni‐*p* = 0.05, multi‐*p* = 0.025), tumor type (uni‐*p* = 0.03, multi‐*p* = 0.036), and 11q13 gene status (uni‐*p* < 0.001, multi‐*p* < 0.001) （Table 3). Additionally, sex (uni‐*p* = 0.005) showed a significance difference in uni‐variate; ENE (multi‐*p* = 0.007) and multi ENE (multi‐*p* = 0.044) showed siginificance differences in multivariate （Table 3). In a subgroup analysis of OS, both uni‐variate and multivariate analyses showed significance differences in the 11q13 gene status (uni‐*p* = 0.032, multi‐*p* < 0.001), histological grade (uni‐*p* = 0.005, multi‐*p* = 0.001), multi ENE (uni‐*p* = 0.033, multi‐*p* = 0.044) and sex type (uni‐*p* = 0.007, multi‐*p* = 0.008) (Table 4).

**TABLE 3:** Univariate and multivariate analysis of DFS.

Characteristics	3‐years DFS rate	Univariate			Multivariate		
		HR	95% CI	*p*	HR	95% CI	*p*
11q13 (amp vs. wild‐type)	21.0% vs. 52.6%	2.445	1.327–4.507	**< 0.001**	4.496	2.171–9.308	**< 0.001**
Age (< 60 vs. ≥ 60)	37.4% vs. 35.1%	0.859	0.458–1.612	0.62			
Sex (male vs. female)	40% vs. 12.5%	0.467	0.158–1.377	**0.005**			
HPV status (negative vs. positive)	38% vs. 27%	1.05	0.479–2.338	0.88			
T grade (T1 vs. T2 vs. T3 vs. T4)	32% vs. 45% vs. 32.4% vs. 35.9%	3.338	0.761–14.64	0.58			
N grade (N0–2 vs. N3)	30.8% vs. 41.4%	1.220	0.665–2.240	0.49			
TNM (III vs. IV)	30% vs. 39.6%	1.429	0.601–3.396	0.35			
Tumor type (1 vs. 2 vs. 3)^a^	54% vs. 42.9% vs. 20%	1.733	1.010–2.973	**0.03**	1.732	1.036–2.897	**0.036**
Histological grade (G1‐2 vs. G3)	47.3% vs. 21.1%	0.583	0.311–1.095	**0.05**	2.203	1.106–4.386	**0.025**
ENE (Negative vs. Positive)	37.9% vs. 36.1%	0.789	0.358–1.735	0.70	3.948	1.445–10.78	**0.007**
Multi.ENE (negative vs. positive)	37.6% vs. 35.6%	0.788	0.357–1.735	0.515	2.635	1.029–6.753	**0.044**
IV.V (negative vs. positive)	31.7% vs. 56.3%	1.388	0.670–2.874	0.39			
Multi region (negative vs. positive)	27.4% vs. 47.2%	1.310	0.722–2.379	0.35			
Treatment (CCRT vs. RT)	52.5% vs. 9.3%	2.208	1.166–4.178	**0.004**	10.05	3.221–31.33	**< 0.001**

^a^ 1: oral cavity; 2: oropharynx; 3: carcinoma of the maxillary sinus.

**TABLE 4:** Univariate and multivariate analysis of OS.

Characteristics		Univariate			Multivariate		
	3‐years OS rate	HR	95% CI	*p*	HR	95% CI	*p*
11q13 (amp vs. wild‐type)	40.3% vs. 66.7%	2.066	1.037–4.119	**0.032**	3.286	1.388–7.380	**0.007**
Age (< 60 vs. ≥ 60)	53.5% vs. 56.1%	0.904	0.432–1.889	0.781			
Sex (male vs. female)	61.0% vs. 0%	0.300	0.063–1.418	**0.007**	4.135	1.428–11.97	**0.009**
HPV status (negative vs. positive)	57% vs. 31%	0.87	0.349–2.213	0.77			
T grade (T1 vs. T2 vs. T3 vs. T4)	51.9% vs. 54.7% vs. 51% vs. 60.7%	0.711	0.387–1.309	0.329			
N grade (N0‐2 vs. N3)	49.3% vs. 55.2%	0.979	0.492–1.951	0.952			
TNM (III vs. IV)	48.9% vs. 54.3%	0.982	0.407–2.366	0.962			
Tumor type (1 vs. 2 vs. 3)^a^	58.6% vs. 53.6% vs. 40%	2.266	1.277–4.02	0.142			
Histological grade (G1‐2 vs. G3)	69.4% vs. 34.1%	0.407	0.193–0.855	**0.005**	3.685	1.657–8.196	**0.001**
ENE (negative vs. positive)	67.7% vs. 45.9%	0.533	0.269–1.058	0.061			
Multi.ENE (negative vs. positive)	61.2% vs. 35.7%	0.504	0.198–1.281	**0.033**	3.292	1.352–8.014	**0.009**
IV.V (negative vs. positive)	56.2% vs. 48.1%	0.919	0.390–2.162	0.839			
Multi region (negative vs. positive)	59.1% vs. 53.7%	1.004	0.507–1.988	0.99			
Treatment (CCRT vs. RT)	56.5% vs. 55.3%	1.250	0.609–2.567	0.515			

^a^ 1: oral cavity; 2: oropharynx; 3: carcinoma of the maxillary sinus.

## 4 Discussion

The prognostic value of expression of 11q13 (FGF‐3/4/19) in local advanced HNSCC remains unclear. This study is the largest sample size of patients with 11q13 mutation so far. We included 35 patients with mutations and 35 matched patients without mutations, and significant differences in DFS and OS were observed (3‐year DFS: 21.0%, 3‐year OS: 46.4%). Multivariate analyses also revealed that 11q13 amplification significantly predicted worse DFS and OS. The amplification group's recurrence pattern revealed a roughly similar percentage of DM and LRR. The 60 Gy radiation field was where the LRR mostly happened.

In the FGF family, FGF2 has been doubly extensively studied in previous studies. In the following year, Forootan et al. studied 51 patients with squamous cell carcinoma (SCC) of the tongue and indicated that squamous cell carcinomas can synthesize bFGF, which has the potential to regulate angiogenesis [21]. In 2014, Bandoh et al. reported on 70 patients with SCC of the maxillary sinus, suggesting that FGF‐2 expression was linked to resistance to radio‐chemotherapy, resulting in a worse prognosis [22]. However, clinical studies specifically investigating the prognostic value of 11q13 (FGF‐3/4/19) expression in HNSCC are limited. The present study confirms that the survival of 11q13 amplification is worse than that of the wild‐type group. The negative impact of 11q13 amplification has been partly explained by its role in stimulating tumor angiogenesis, leading to the hypothesis that 11q13 expression may also be associated with poorer prognosis in HNSCC patients [23]. Previous studies have shown that 11q13 amplification negatively correlates with PD‐1 inhibitor therapy response in a retrospective clinical cohort [20]. These genetic alterations may serve as potential predictive biomarkers to identify patients who are unlikely to benefit from immunotherapy. Moreover, previous studies have indicated that CCND1 protein expression in 11q13 is linked to poorer survival outcomes [24]. Despite the prognostic indicator, the development of small molecule drugs targeting FGFR has garnered significant attention from pharmaceutical chemists, driving rapid advancements in both the understanding of their mechanisms of action and their clinical applications [19]. These progress provides a solid theoretical foundation for the precision medicine and personalized treatment strategies that are widely used today.

Of the 31 patients with 11q13 amplification LA‐HNSCC who underwent PORT in this study, 28 were oral cavity cancer (OCC). Previous studies have shown that patients with OCC have a distinctive pattern of recurrence, with a predominance of localized recurrence, which may reflect the more aggressive biology of patients with OCC and their resistance to chemotherapy and radiotherapy [25]. Previous genomic data have begun to reveal subgroups of oral cancers with distinct molecular profiles and unique molecular drivers, including a subgroup of CASP8 (OMIM 601763) patients with or without FAT1 (OMIM 600976) mutations [26, 27]. This is corroborated by the recurrence pattern of LRR in the 11q13 patients included in this study and suggests that 11q13 amplification may also be one of the molecular bases of the recurrence pattern in OCC patients. The recurrence pattern in OCC occurs predominantly in the 60 Gy irradiation range. Therefore, modifying postoperative adjuvant treatment is also important, suggesting that local simultaneous integrated boost (SIB) may be necessary for these patients.

The shortcoming of this study is that the sample size is not large enough. In addition, no significant correlation was found between the frequency of 11q13 amplification and the length of survival, and no correlation was found with various clinical indicators. Further expansion of the sample size or prospective studies is needed in the future to verify the findings of this paper.

## 5 Conclusion

LA‐HNSCC patients with 11q13 amplification exhibited significantly worse DFS and OS compared to wild‐type patients. The recurrence pattern in patients with 11q13 amplification was primarily characterized by in‐field recurrences within the 60 Gy dose.

## Author Contributions

**Chang Jiang:** investigation, writing – original draft, writing – review and editing, methodology, software, data curation, formal analysis, project administration, visualization. **Shengjin Dou:** conceptualization, validation, writing – review and editing, data curation, supervision. **Yu Wang:** project administration, resources, data curation, validation, visualization. **Wen Jiang:** resources, project administration. **Lulu Ye:** investigation, validation, formal analysis. **Rongrong Li:** investigation, validation, formal analysis. **Xiaolong Fu:** project administration, funding acquisition, visualization, validation. **Lin Zhang:** funding acquisition, formal analysis, project administration. **Guopei Zhu:** funding acquisition, conceptualization, project administration, investigation, supervision.

## Ethics Statement

The Study Conducted in accordance with the principles of the Declaration of Helsinki of the World Medical Association, with the endorsement of the Shanghai Ninth People's Hospital Ethics Committee, and with the informed consent of all patients. Ethics approval number: SH9H‐2024‐T124‐1.

## Conflicts of Interest

The authors declare no conflicts of interest.

## Supporting information


**Data S1:** Supporting Information

## Data Availability

The data that support the findings of this study are available on request from the corresponding author. The data are not publicly available due to privacy or ethical restrictions.
