# Supplementary material for: Prognosis and Failure Patterns of 11q13 Amplified Local Advanced Squamous Cell Carcinoma of the Head and Neck
Source: Cancer Med. 2025 Sep 20;14(18):e71235. doi: 10.1002/cam4.71235 (PMC12449658; doi:10.1002/cam4.71235)
Supplement: Supplementary file 1 — Data S1: Supporting Information [file CAM4-14-e71235-s001.docx]

**Supplement**

Supplementary Materials

The number of 11q13 amplification patients was initially determined by NGS The amplification group used propensity score matching (PSM) at a 1:1 ratio to matched with patients from the control pool, which including 11q13 NGS negative patients and 11q13 unknown patients. CCND1 amplification was tested by FISH for 11q13 unknown patients, and those who were negative enrolled in the 11q13 wild-type group, otherwise they were added to the amplification group. In the meantime, the new 11q13 amplification patients were 1:1 PSM with the control pool patients again, and undergone pathologically confirmed. Logistic regression was used to generate the propensity scores, adjusting for baseline factors such as age, sex, tumor site, TNM stage, N category, and treatment. The baseline characteristics of the two groups were compared before and after matching using Fisher's exact test or the chi-square test.
